# New Method for Quantification of Phenotypic Plasticity Reveals How Plasticity Changes Over Time in the Diatom *Thalassiosira weissflogii*

**DOI:** 10.1002/ece3.73072

**Published:** 2026-02-17

**Authors:** Lilian Hoch, Andrei Herdean, Stephen Woodcock, Kittikun Songsomboon, Breanna Osborne, Peter J. Ralph

**Affiliations:** ^1^ Climate Change Cluster University of Technology Sydney Ultimo New South Wales Australia; ^2^ School of Mathematical and Physical Sciences University of Technology Sydney Ultimo New South Wales Australia

**Keywords:** diatoms, phenomics, phenotypic plasticity, photobiology, trait‐based ecology

## Abstract

Quantifying phenotypic plasticity, the capacity of organisms to adjust phenotypes in response to environmental changes, is essential for understanding ecological and physiological resilience under climate stress. However, existing methods often lack flexibility and precision, especially under multi‐dimensional environmental conditions. Here, we introduce a novel statistical approach, the Environmentally Standardized Plasticity Index (*ESPI), which integrates Hedges' *g* for effect size quantification and Euclidean distance for characterizing environmental variability. We validated this method using both simulated datasets and empirical data from the marine diatom *Thalassiosira weissflogii* , investigating five key phenotypic traits over 7 days under varying temperature, irradiance, and nutrient conditions. Our findings indicate distinct temporal patterns of plasticity: certain traits, such as photosynthetic efficiency (alpha) and saturation irradiance (*E*_k_), demonstrated high initial plasticity followed by gradual acclimation, whereas others, like pigment composition, exhibited delayed phenotypic responses. This temporal dimension highlights the critical role of the growth phase in shaping plasticity responses. The proposed *ESPI method provides a robust, intuitive, and versatile framework for quantifying phenotypic plasticity, offering significant advances in predicting organismal adaptation to environmental change.

## Graphical abstract

Quantifying phenotypic plasticity, the capacity of organisms to adjust phenotypes in response to environmental changes, is essential in understanding ecological and physiological resilience under climate stress. Here, we introduce a novel statistical approach, the Environmentally Standardised Plasticity Index (*ESPI), which integrates Hedges'*g* for quantification of the effect size and Euclidean distance for characterizing environmental variability. Our findings indicate distinct temporal patterns of plasticity in the marine diatom *Thalassiosira weissflogii*.

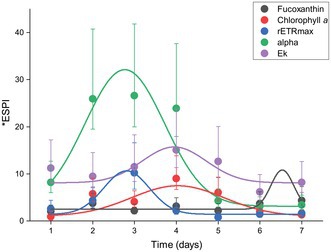

## 1 Introduction

Phenotypic plasticity, the ability of organisms to alter their phenotype in response to environmental changes, is a crucial adaptation mechanism in phytoplankton, microalgae, and cyanobacteria. This plasticity can manifest in various ways, such as changes in carbon fixation rates (Ji et al. 2020), pigmentation (Stomp et al. 2008), and morphology (Lin et al. 2020; Morales and Trainor 1997), among other traits. Plasticity can vary among species and is influenced by environmental variation (Leung et al. 2020). Phenotypic plasticity plays a significant role in competition and adaptation to fluctuating environments and can contribute to changes to community functional responses (Beier et al. 2015). Understanding phenotypic plasticity is crucial for predicting species' responses to environmental changes and their ecological response (Ji et al. 2020).

There is a significant need for a consensus on, or at least a clearer understanding of, the methods used to study phenomes and their plasticity within environmental research. Many current studies vary widely on a philosophical level, focusing on different aspects of the genotype‐to‐phenotype pipeline. This results in different attention on traits, environmental contexts, and statistical methods, which can make any type of cross‐comparison between species impossible. This lack of methodological consistency contributes to a broader gap in our understanding of how phenotypes, shaped by complex interactions between genes and environments, can be reliably studied and compared (Furbank and Tester 2011; Houle et al. 2010; York 2019). To further the field of phenomics, as to better address the phenome genome gap which continues to emerge because of increasing accessibility of genetic sequencing (Furbank and Tester 2011; Yang et al. 2009), greater coordination is required across the methodological approaches. This includes clear reporting of environmental conditions past and present, as well as trait selections, which may differ based on research goals but must be clearly stated and justified.

Under anthropogenic climate change, it is vital to establish a clear picture of how certain species will fare based on not only their current patterns of behaviour but also on their intrinsic capacity for phenotypic plasticity (Nicotra et al. 2010; Sackett et al. 2013). However, consensus is still lacking on how plasticity should be defined and quantified. This challenge has persisted for decades, reflecting both limited knowledge of underlying biological mechanisms and the statistical difficulties associated with capturing plastic responses (Laitinen and Nikoloski 2019; West‐Eberhard 2005; Argyle et al. 2021; Pélabon et al. 2020; Valladares et al. 2006). Streamlining statistical methods is the key aspect of this research which will allow for greater cross‐comparison.

To help reconcile these methodological discrepancies and facilitate a stronger understanding of multi‐trait plasticity, we introduce a novel statistical framework to unify existing approaches under a single, flexible methodology. This framework adapts and extends the Environmentally Standardized Plasticity Index (ESPI) proposed by Valladares et al. (2006), allowing researchers to characterise phenotypic plasticity even under complex experimental conditions quantitatively. The method has been revised on the basis that the original ESPI is scale dependent, meaning that cross‐trait and cross‐species comparison becomes difficult, and is inherently limited to one environmental axis. The revised method allows for cross‐trait comparison at a given number of environmental variables, allowing for more effective comparison between and across species. A trait‐wise approach to plasticity will likely be the most appropriate as to maintain nuance when looking at a phenotype, rather than overprocessing the data in the hopes of producing a singular ‘whole phenotype’ value.

Here, ESPI is adapted to study quantitative phenotype plasticity and validated using a simulation dataset and a case study on the diatom *Thalassiosira weissflogii* . Aside from computing plasticity according to *ESPI, each trait is examined on a day‐by‐day basis to resolve how plasticity in the diatom behaves on a temporal scale. This allows for a deeper understanding of how phenotypic plasticity behaves after cultures experience an environmental shift and could be a tool employed to gain an understanding of the mechanistic drivers of plasticity in future study.

This in vivo study of the diatom's plasticity incorporates three environmental factors which are relevant under anthropogenic climate change: temperature, light, and nutrient availability. Sea surface temperatures as well as top 2000‐m ocean temperatures have reached unprecedented highs in recent years and significantly impact microorganism populations and distribution, a trend that will not slow in the coming decades (Cheng et al. 2023, 2025). Changes in sedimentation levels combined with the destabilisation of global currents, largely due to decreased ice cover in the poles and extreme weather events, have a significant effect on irradiance levels by dysregulating stratification (Marinov et al. 2010). Nutrient availability and aqueous carbon concentration are additionally severely impacted by human activity and climate change, including nitrogen and phosphate cycling which have both undergone major increases from preindustrial times (Falkowski et al. 2000; Field et al. 1976; Jiang et al. 2023). These environmental factors are part of a larger picture of interwoven drivers which will likely permanently alter the dynamics of the world's oceans. Algae play a foundational role in these dynamics, highlighting the need for multidriver phenotypic analysis such as is proposed here. This study aims to provide a flexible framework and associated method by which the outcomes of climate change on phenotypic expression, understood to be plasticity, can be quantified on a trait level.

### 1.1 Statistical Theory

#### 1.1.1 New *ESPI Calculation

The original ESPI equation (Equation 1; Valladares et al. 2006), which sets 𝑋 and 𝑥 as the maximum and minimum phenotypic values, and 𝐸 and 𝑒 as their corresponding environmental values, lacks any statistical differentiation between these extremes, preserves trait scales, and does not extend to multi‐dimensional environmental gradients. As the equation utilized raw data scales, it is not appropriate for cross‐trait comparisons.

(1) $$\text{ESPI}=\frac{X-x}{E-e}$$

To resolve these limitations, the new proposed formula for calculating the *ESPI integrates Hedges' *g* in the numerator to quantify the effect size between two sample groups and Euclidean distance as the denominator, reflecting environmental change.

(2) $$*\text{ESPI}=\frac{\text{Hedges}\;g}{\text{Euclidean distance}}$$

The numerator in this instance is proposed to be Hedges' *g* (also known as unbiased Cohen's *d*). The ‘*d*’ statistic (Cohen 1992, 1969) quantifies effect size through pairwise comparison of standardized means, but tends to overestimate variance in small sample groups, as seen in this application. Hedges' *g* (Hedges 1981) corrects for bias in small sample sizes, making it more reliable than Cohen's *d* in such contexts, and is applied as a correction factor to the base formula.

**FIGURE 1:**
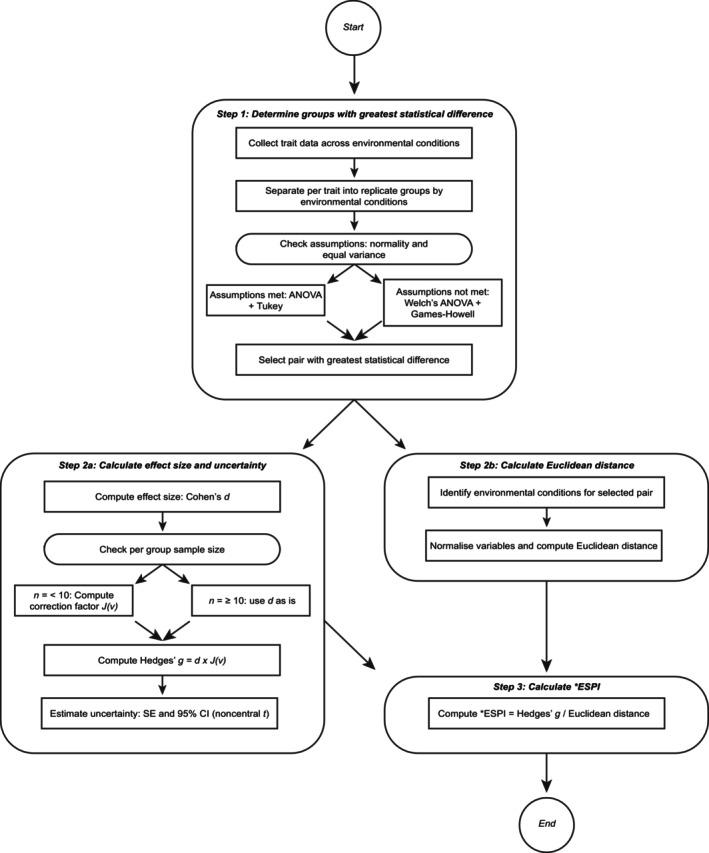
Flowchart depicting the method employed for the calculation of *ESPI.

Before the calculation of Hedges' *g* it is still necessary to test significance through parametric and non‐parametric testing of the null hypothesis; however, this should be treated as an intermediary step and will produce an independent result (Figure 1, Step 1). Traditional *t* tests can assess whether an effect is statistically significant but do not provide a direct measure of effect size. Thus, additional effect size metrics are necessary to quantify the magnitude of phenotypic shifts, as such *p* values cannot reveal the strength of a relationship in adequate detail (Goulet‐Pelletier and Cousineau 2018). Here, statistical difference forms a baseline measurement in this application to the *ESPI. The pairwise comparison with the greatest statistical difference is then used to calculate variance in the subsequent steps. This can be computed even if there is no statistically significant difference according to the applied threshold; the greatest difference in the observed dataset can still be used to calculate Hedges' *g*. Once Hedges' *g* is calculated, its standardized nature allows for meaningful comparisons, preventing artificially inflated *ESPI values arising from differences in the scales of measurement between different traits. Furthermore, the interpretation is intuitive: larger values of Hedges' *g* indicate more substantial phenotypic shifts, while smaller values correspond to weaker effects.

The base formula of Cohen's *d* (Figure 1, Step 2a; Equation 3) uses ${\bar{x}}_{1}$ and ${\bar{x}}_{2}$ as the numerator (means of sample groups) and the denominator *S* (SD).

(3) $${\text{Cohen}}^{\prime }s\;d=\frac{{\bar{x}}_{1}-{\bar{x}}_{2}}{S}$$

Pooled standard deviation is widely agreed to be the most appropriate measure when comparing two independent sample groups for Cohen's *d* (Goulet‐Pelletier and Cousineau 2018) (Equation 4). *n*_1_ and *n*_2_ are the sample sizes of groups 1 and 2, respectively, and *S*_1_ and *S*_2_ are the associated standard deviations of these groups. This accounts for each group's sample size (*n*), which may vary between groups (perhaps due to error or outlier removal). The term is generally expressed as ‘*d*_*p*_’ or ‘*d*_*s*_’ (Goulet‐Pelletier and Cousineau 2018).

(4) $$S_{\text{pooled}}=\sqrt{\frac{\left(n_{1}-1\right)S_{1}^{2}+\left(n_{2}-1\right)S_{2}^{2}}{n_{1}+n_{2}-2}}$$

Next, the Cohen's *d* can be transformed to Hedges' *g*. Cohens' *d* is found to overestimate effect size, with a relatively large bias occurring with sample sizes of less than 10 in each group, or a total of less than 20 (Wasserman et al. 1988). When sample groups total more than 20, this step can be avoided and Cohens' *d* used as the numerator in *ESPI, though to avoid ambiguity referred to as *g* since the correction factor can be assumed to be ~1. The correction factor (*J*(*v*)) must be determined based on the dataset (Equation 5). A simplified method is presented as the extended formula requires use of the Gamma function (Γ), which can be computationally demanding (Equation S1). The approximated correction factor (Equation 5) allows for a simplified calculation and can be used for sample sizes over 10. This is represented by *v* in the equation is the total sample size number across the two groups minus 2 (when a two‐group design is used; however, more options exist for repeated measure designs).

(5) $$J\left(v\right)\approx \left(1-\frac{3}{4v-1}\right)$$

In the following simulations, *v* = 10, *J*(*v*) is 0.9227 and the approximation of *J*(*v*) is 0.9231, making the difference in the final correction factor near negligible; however, the extended formula for correction factor calculation will be applied for demonstration purposes in the following datasets (Equation S1). Once the correction factor has been applied, Hedges' *g* can be calculated by multiplication of Cohen's *d* and the correction factor:

(6) $$g=d\times J\left(v\right)$$

The final value obtained by Equation (6) can be implemented into the *ESPI formula.

#### 1.1.2 Calculation of Euclidean Distance

The denominator in Equation (2), Euclidean distance, calculates the distance between two points in any number of dimensions, and has been used to quantify distances in environmental space (Step 2b, Figure 1) (Champely and Chessel 2002; Kietpawpan et al. 2003). Functionally, this means that Euclidean distance and environmental change are positively correlated. Here, each environmental variable is normalized on a 0 to 1 scale within the experiment, providing a scale‐invariant measure of Euclidean distance. Note that Euclidean distance is often denoted as *d*; however, to avoid confusion with Cohen's *d* it will be referred to by its name. Here, the Euclidean distance across *n* dimensions is calculated using Equation (7). In this equation, *i* signifies each dimension, and *q* and *p* are the coordinates of the two points being examined, meaning *q*_*i*_ and *p*_*i*_ are the coordinates of two points in the *i*th dimension.

(7) $$\text{Euclidean distance}=\sqrt{\Sigma_{i=1}^{n}{\left(p_{i}-q_{i}\right)}^{2}}$$

#### 1.1.3 Standard Error and Confidence Intervals

In understanding the strength of the *g* value under this model, standard error (SE) and confidence intervals (CI) can and should be calculated (Figure 1, Step 3). Because the variance is calculated based on two distinct means, unless sample sizes are very large, it will follow a non‐central *t* distribution (Goulet‐Pelletier and Cousineau 2018), unless a central *t* distribution can be proven. The method known as ‘true’ standard error (Equation 8) (Hedges 1981) remains to be the most appropriate measure when looking at sample sizes under 50.

(8) $$\mathrm{SE}=\sqrt{\frac{v}{v-2}\;\frac{2}{\begin{array}{c} \sim \\ n \end{array}}\;\left(1+\delta^{2}\frac{\begin{array}{c} \sim \\ n \end{array}}{2}\right)-\frac{\delta^{2}}{{\left(J\left(v\right)\right)}^{2}}}$$

In this equation for calculation of ‘true’ standard error, *J*(*v*) is the correction factor, $\tilde{n}$ is the harmonic mean of the *n* of the two sample groups, *v* represents the total sample number across the two groups, and δ is the true standardized effect size in the population (which is replaced by the effect size estimate *g* as previously calculated, as true effect size is unknown).

Confidence intervals can be calculated to add strength and ensure the result is robust, assessing suitability and precision. Following on from the assumptions previously taken, the non‐central *t* method is most appropriate (Equations 9 and 10), though central *t* distribution and bootstrapping are sometimes also used. The non‐centrally parameter (*λ*) is obtained with *g* representing the effect size, and $\tilde{n}$ as the harmonic mean of the *n* of the two sample groups (Equation 10).

(9) $$\lambda_{\text{between groups}}=g\sqrt{\frac{\begin{array}{c} \sim \\ n \end{array}}{2}}$$

After this, *λ* can be applied to calculate CIs (Equation 10) in which *λ* is obtained by the previous formula, *t*_L_ is the lower limit, and *t*_U_ is the upper limit. For a typical 95% symmetric confidence interval, these would be the 2.5th and 97.5th percentiles of the distribution. Square brackets denote that the extremity of the intervals is inclusive. *g*_L_ and *g*_U_ denote the lower and upper limits of the *g* statistic using *t*_L_ and *t*_U_ as the lower and upper limits of the *λ* interval.

(10) $${\mathrm{CI}}_{g}=\left[g_{L}=t_{L}÷\frac{\lambda }{d},g_{U}=t_{U}÷\frac{\lambda }{d}\right]$$

#### 1.1.4 Method Implementation

To demonstrate the calculation of phenome plasticity using the amended *ESPI a sample dataset is analysed from the model diatom *T. weissflogii*. This species of diatom has widely been studied due to its use in aquaculture, ecological importance, biotechnological applications and in being the first diatom to undergo genome sequencing (Alverson et al. 2011; García et al. 2012; Marella and Tiwari 2020). In this experiment, 48 96‐well plates were inoculated with culture media under 24 different environmental conditions, and daily trait measurements were performed. Temperature, irradiance, and nutrient concentration were altered to induce various phenotypic responses.

**FIGURE 2:**
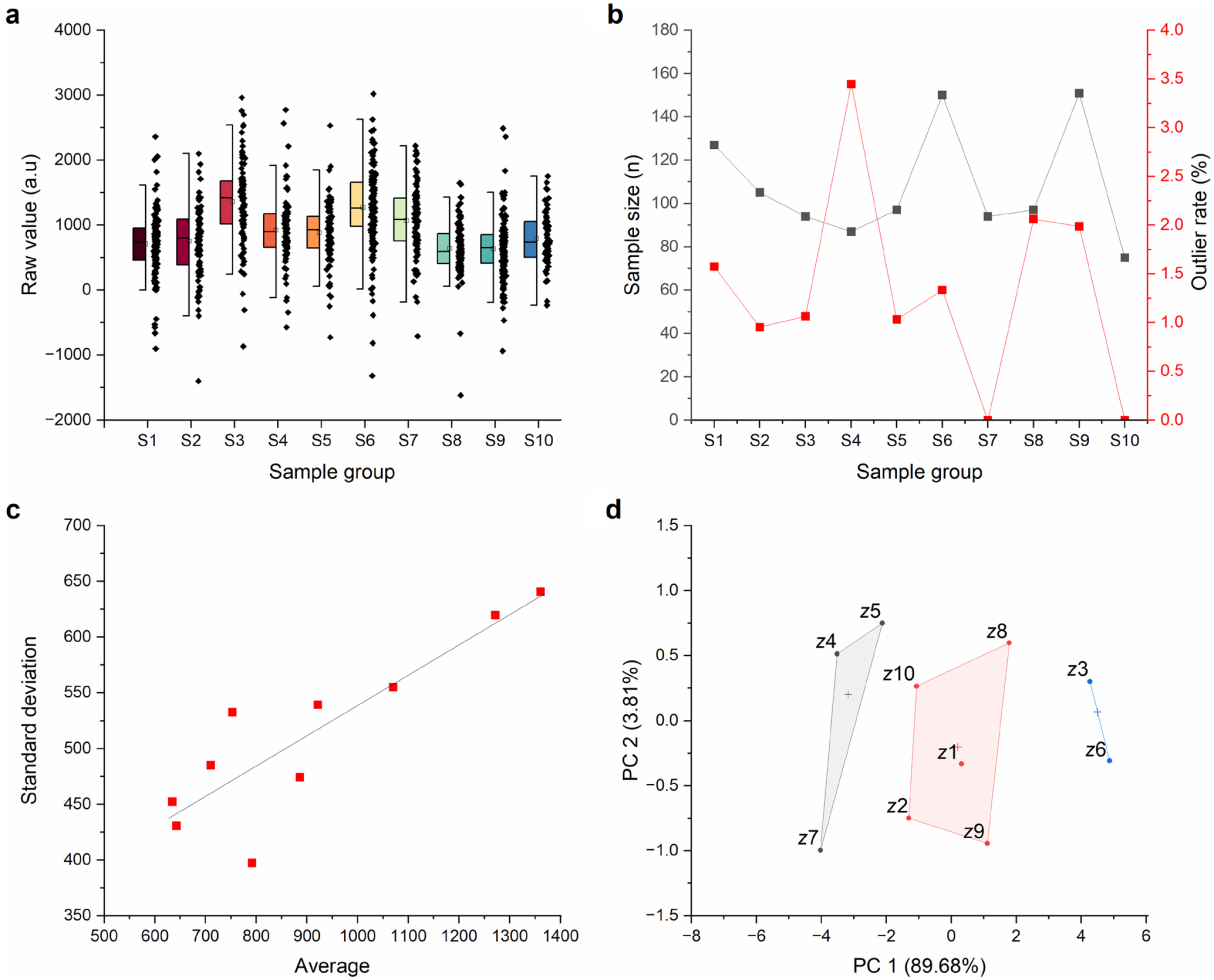
Simulation data and dataset features. (a) Half box plot depicting raw data values in the simulation series. S1–S10 represent the 10 sample groups. Boxes indicate interquartile range, whiskers denote outlier boundaries (based on interquartile range multiplied by 1.5), and mean value is shown by the bar in each box. (b) Sample size (left *y*‐axis) and outlier rate (right *y‐*axis) per sample group (S1–S10). (c) Heteroscedasticity of the raw dataset. Standard deviation (*y*‐axis) and average (*x*‐axis) of each of the 10 sample groups are plotted. Linear regression demonstrates the positive mean–variance trend. (d) Principal component analysis with *k*‐means clustering (*k* = 3) of the 10 environmental variables (*z*1–*z*10) assigned to across each dataset (S1–S10). Three correlated clusters emerge; *z*4, *z*5, and *z*7 in grey; *z*10, *z*8, *z*1, *z*2, and *z*9 in red; *z*3 and *z*6 in blue.

Additionally, simulated data were prepared by creating 10 sample groups under different environmental pressures, each with a certain number of ‘replicates’. The simulation data was designed to mimic a real life ‘messy’ dataset, to appropriately stress test the model. Sample sizes were imbalanced, drawn from log‐normal distributions, with random missing data and outliers built in through heavy‐tailed errors and contamination (Figure 2a,b). Additionally, the data were made heteroscedastic, so that groups producing larger means also had greater variance (Figure 2c). ‘Environmental values’ were also generated with the intention of mimicking real‐world interactions. A set of 10 environmental factors (*z*1–*z*10) was generated for each group of data. In this, correlation was imposed through a Gaussian copula, so that axes display a degree of interdependence, as would be seen in ecological examples (i.e., Colder temperatures correlate with greater bioavailability of nutrients, Harrison and Cota 1991). Correlation of environmental variables can be seen through *k*‐means clustering PCA in Figure 2d. This dataset was used in the comparison of the original ESPI method as per Valladares et al. (2006), and the new *ESPI method proposed in this paper.

## 2 Materials and Methods

### 2.1 Culture Maintenance

*Thalassiosira weissflogii* strain CS‐871 was maintained under 20°C in standard F/2 media supplemented with silica (Guillard and Ryther 1962). Culture was upscaled to produce adequate inoculum at mid‐exponential phase, upon which experimental plates were inoculated.

### 2.2 Experimental Design

Forty‐eight 96‐well plates (Corning, Corning NY) were prepared with three concentrations of media with varied concentrations of nutrients. Media types were prepared for a final concentration of 50%, 100%, and 200% standard F/2 in 300 μL (Algaboost, AusAqua SA). Rows in well plates were filled with 250 μL of one of three media types in ascending alternation, producing 32 replicates per plate (using Opentrons OT‐2, Opentrons NY). Well plates were then inoculated with 50 μL of *T. weissflogii* in each well. Of the 48 well plates, 8 sets of 6 replicates were made which would then be incubated under varying temperature and light conditions, as seen in Table S1. This brought the total number of replicates per unique environmental condition to 192, across 24 unique environmental conditions. Control conditions were included in the matrix. Day 0 measurements were taken, and cultures were subsequently incubated for 7 days in the assigned conditions.

### 2.3 Spectroscopy

Optical density was taken at 480 nm excitation and 550 nm emission, and 480 nm excitation 680 nm emission to provide rough estimates of secondary photosynthetic pigments (mainly fucoxanthin) and chlorophyll *a*, respectively (Spark Cyto Tecan Trading AG, Switzerland). This was repeated for each of the 48 plates every day.

### 2.4 Flow Cytometry

Each day, 5 wells were selected from 24 of the 48 plates so that 9 replicates per unique condition could be analysed using flow cytometry (Cytoflex LX, Beckman Coulter USA). Cell counts per mL were used to calculate growth for the duration of the experiment as a quality control and to eliminate cultures from the experiment that collapsed after inoculation of plates.

### 2.5 Photobiology

Pulse amplitude modulated (PAM) fluorometry was performed daily using a Flat FluorCam FC 1300/2020 (PSI, Drasov, Czech Republic). Rapid light curves obtained from these measurements enabled the calculation of rETRmax, alpha, and *E*_k_.

### 2.6 Statistical Analysis

Data collected was arranged and tested for distribution and normality conditions; pairs which elicited the greatest statistical differences were isolated as identified by Welches ANOVA and post hoc Games‐Howell test. From these pairs, Hedges' *g* was calculated according to the methods described earlier and *ESPI was calculated. ChatGTP‐4o (OpenAI) was used to aid in writing the R code used to conduct statistical analysis.

## 3 Results

### 3.1 Simulation Dataset

The simulation dataset was designed to evaluate the benefits and limitations of the proposed *ESPI method. Figure 2a–d ++shows the features of the dataset, of which there are 10 ‘sample groups’ (S1–S10), and the correlations among the environmental factors used (*z*1–*z*10). Raw data scale ranges from −1617 to 3021, with large SDs (Figure 2c) and frequent outliers (Figure 2b). Sample sizes range from 75 to 151 based on outlier rate (Figure 2b), and the heteroscedastic relationship shows an increase in mean as correlated with an increase in standard deviation (Figure 2c). Figure 2d shows the three clusters of correlated environmental factors; *z*4, *z*5 and *z*7 in grey; *z*1, *z*2, *z*8, *z*9, *z*10 in red; and *z*3, *z*6 in blue.

**FIGURE 3:**
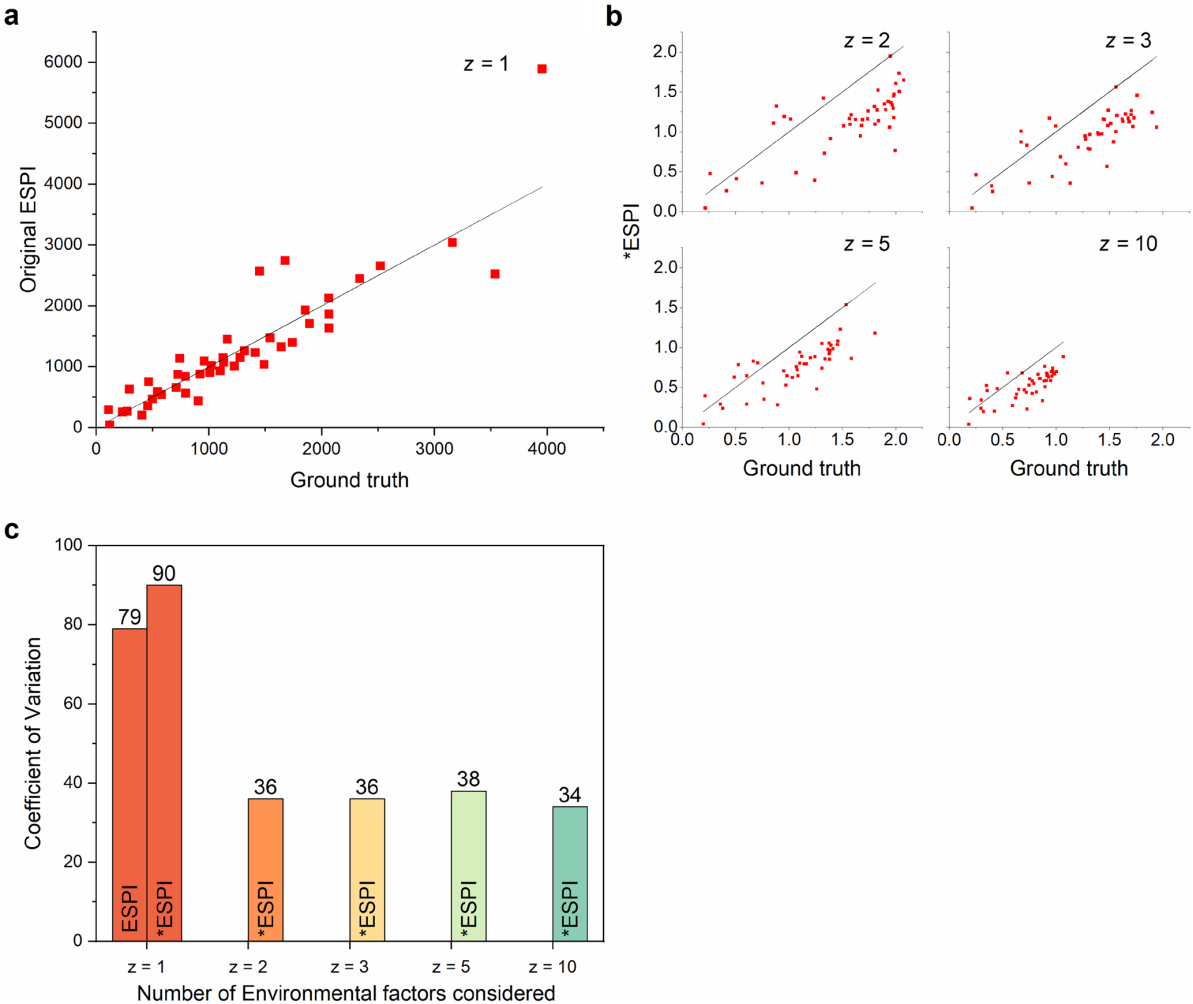
Relationship between original ESPI and *ESPI and ground truth values. (a) Relationship between original ESPI and ground truth standardized effect using a single environmental axis (*z*=1) for each dataset pair in the simulation (45 pairs). (b) Relationship between *ESPI and ground truth standardized effect. *ESPI was calculated for each pair of datasets (S1–S10 producing 45 pairs). Plots represent estimation versus truth when Euclidean distances of 2, 3, 5, and 10 environmental factors are considered. Ground truth was calculated through true means and true pooled standard deviation as determined by the simulation. 1:1 line represents a perfect calibration of the model. Degree of numerical variance indicates sensitivity. (c) Coefficient of variation as calculated for original ESPI using one environmental axis (*z*=1), and for *ESPI using each 1, 2, 3, 5 and 10 environmental axes (*z*=1, 2, 3, 5, 10).

When investigating the relative strength of original ESPI versus *ESPI, pairwise values were computed between all datasets resulting in 45 individual values. ESPI was calculated using *z1* only as per the original method, and *ESPI was calculated using Euclidean distance of 2, 3, 5, and 10 environmental variables. For each combination, a ground‐truth plasticity was computed based on the true mean and the pooled variance. When plotting against ground truth, the original ESPI (Figure 3a) retained its raw value magnitude but adhered more closely to the 1:1 line. *ESPI (Figure 3b) maintained a more comparable scale even as the number of environmental variables increased, but showed a degree of systematic negative bias, ranging from −0.42 to −0.21 as environmental axes increased (Figure S1c).

**FIGURE 4:**
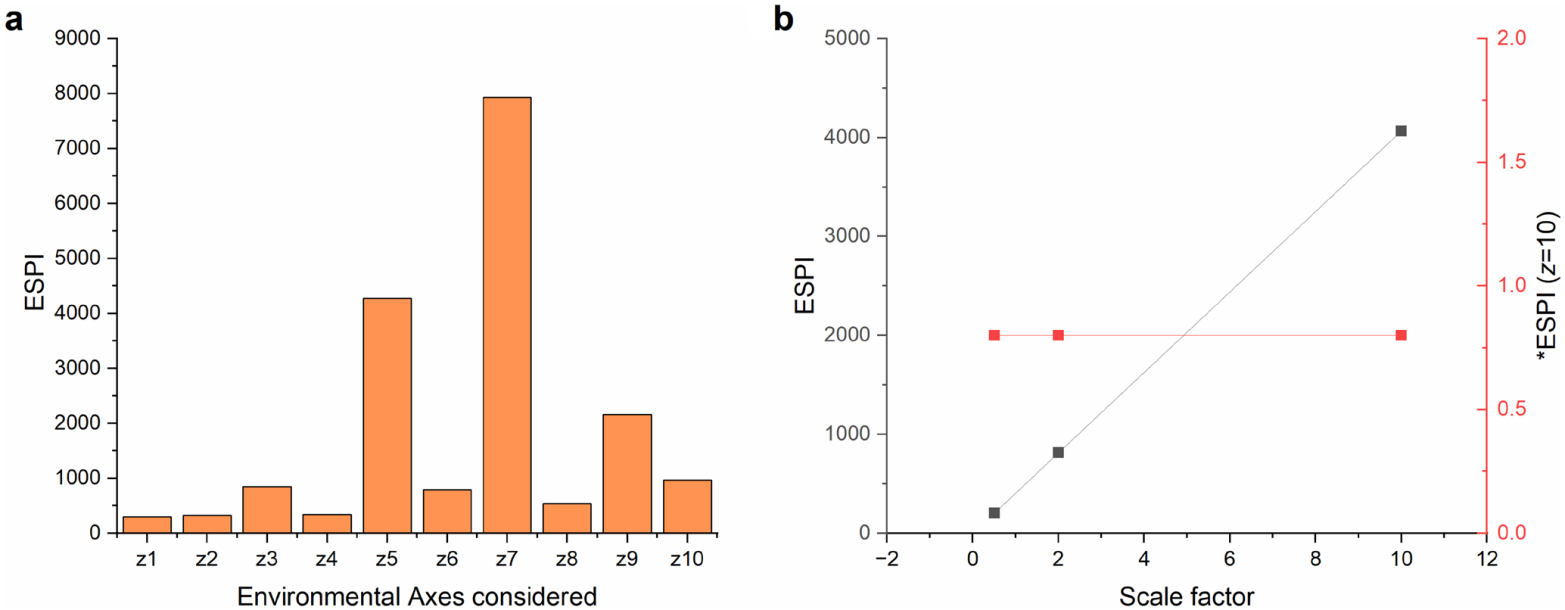
Axis and unit sensitivity of ESPI versus *ESPI. (a) Denominator scale sensitivity of ESPI versus *ESPI. ESPI of s1 and s10 when each of the environmental variables are considered as the single denominator. *ESPI is shown for reference, where Euclidean distance across all 10 environmental parameters is used. (b) Unit sensitivity of ESPI vs. *ESPI. Raw data values were scaled by 0.5, 2, and 10 and ESPI calculated using *z*1 as the one‐dimensional denominator. Mean of all (*n* = 45) ESPI pairs is reported for each scaled point.

Figure 3c displays the coefficient of variation (CV) of ESPI versus *ESPI, as calculated across the same 45 pairwise comparisons of each dataset under the relevant number of environmental factors. ESPI has a slightly lower CV of 79% as opposed to *ESPI under a singular dimension, for which the CV is 90%. Once multiple environmental factors are considered, CV for *ESPI drops to 36%, 36%, 38%, and 34%, respectively. The effect of the environmental factor used on the same dataset is then applied in Figure 4a, when the original ESPI is calculated using each *z* variable. Finally, Figure 4a presents individual axis sensitivity of ESPI, and Figure 4b demonstrates unit sensitivity scaling raw values by 0.5, 2, and 10 and then calculating mean ESPI and *ESPI (*n* = 45). Original ESPI is resultingly calculated as 203.4, 813.6, and 4068 based on scaling, whereas *ESPI remains stable across each level.

### 3.2 Application for Quantification of *T. weissfloggi* Phenotype Plasticity

**FIGURE 5:**
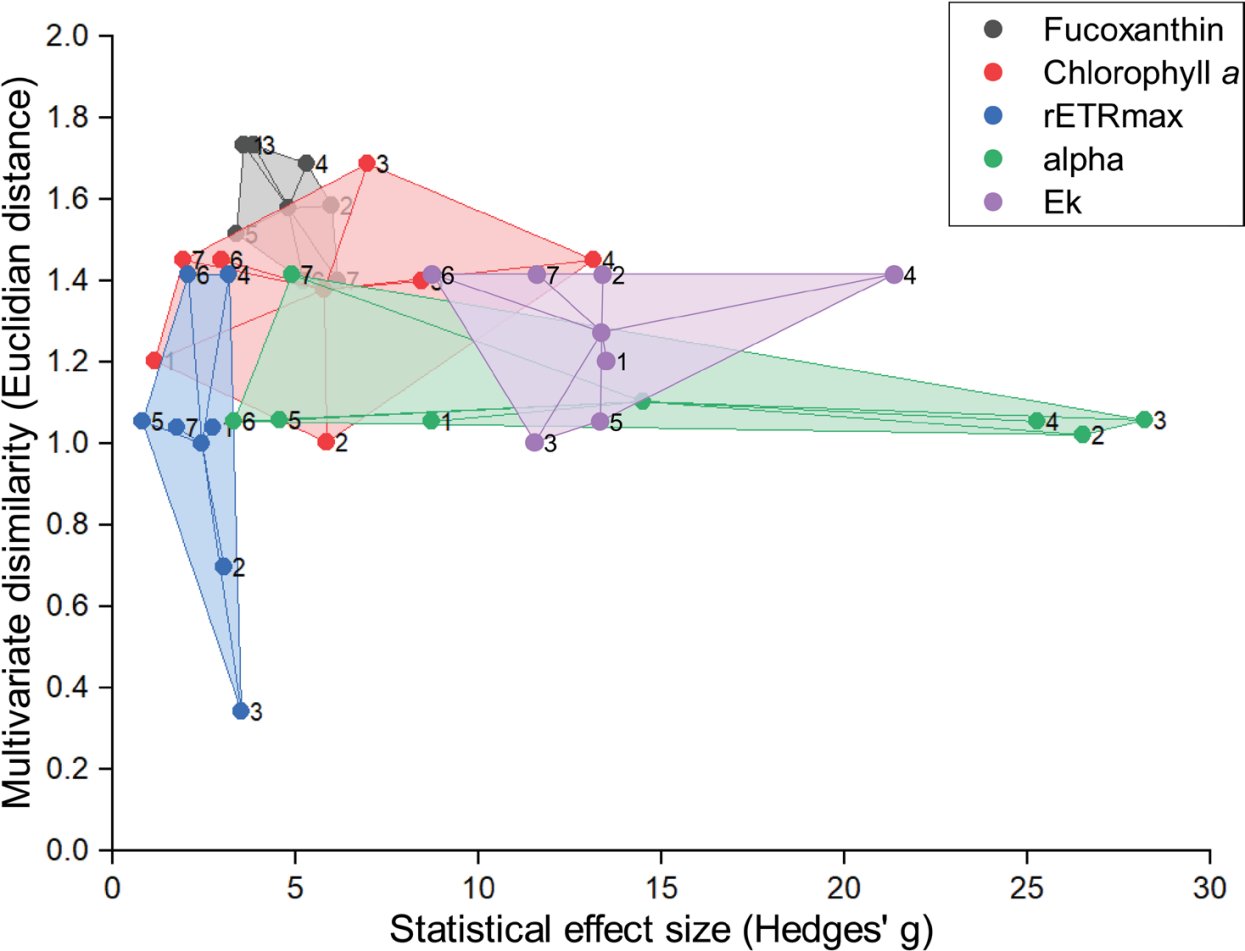
Relationship between statistical and multivariate differences in trait responses of *Thalassiosira weissflogii*. Horizontal axis shows Hedges' *g* calculated between two groups of phenome responses with the greatest statistical difference determined by Welches ANOVA and *post hoc* Games–Howell test. Vertical axis is the Euclidean distance of the two points at which the greatest difference was found in three‐factor space representing the difference between the two environments invoking said phenome response. Color of individual point represents the trait, and each time point is labelled with the day (1–7 days) on which the data was collected. The colored area between the points is the Convex Hull and its centroid.

In Figure 5, the Euclidean distance versus Hedges' *g* of each trait is monitored in *T. weissflogii* on each day of the experiment. It can be observed that as variance shifts, so does environmental change between the points eliciting these responses. Alpha and *E*_k_ show the highest points (g of 28.2 on Day 3 and 11.6 on Day 4, respectively) on both axes for multiple days, particularly on Days 3 and 4. rETRmax shows its highest point on both axes on Day 3 (g of 3.5), while other days show smaller variance and Euclidean distance. Chlorophyll *a* and fucoxanthin appear around the centre of the *x*‐axis (indicating variance); however, chlorophyll *a* appears relatively higher in relation to the *y*‐axis (Euclidean distance). Days 5 through 7 show a trend of clustering at low Hedges' *g* and low Euclidean distance.

**FIGURE 6:**
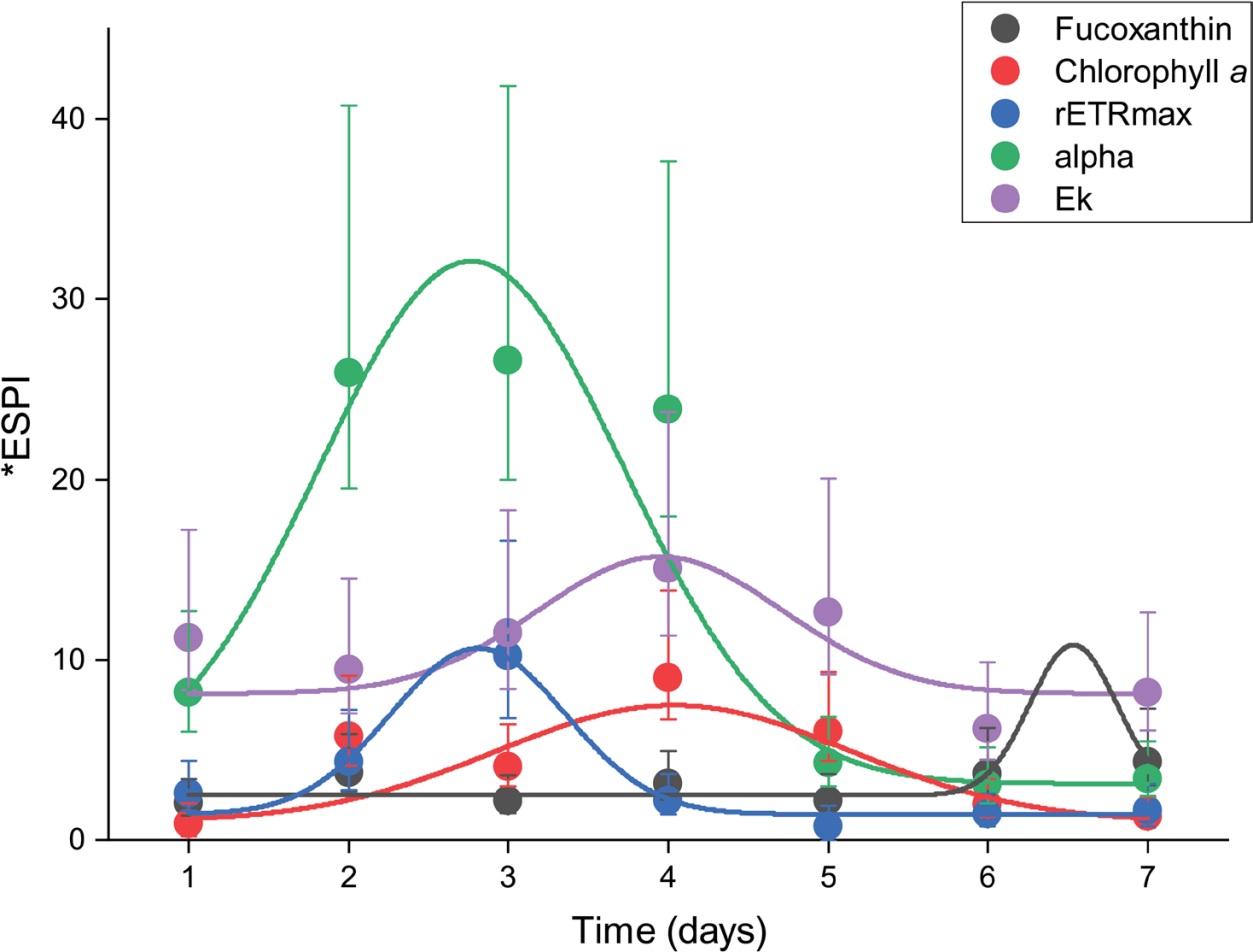
Phenotypic plasticity as a function of time. *ESPI for phenome responses of the five traits in *Thalassiosira weissflogii* with confidence intervals. Data was fitted with a Gaussian function and shows how the plasticity of each trait changes as a function of time. CIs of Hedges' *g* are included with each ESPI.

Across the span of the experiment, *E*_k_ maintains a consistently high effect size with an *ESPI on Day 1, 13.5 to 11.6 on Day 7 (Figures 5 and 6). On Day 7, this occurs under a larger Euclidean distance of 1.41 as compared with 1.2. The highest *ESPI occurs on Day 4, after which a drop occurs. Alpha shows a high variance on day one at 8.71, the second highest to *E*_k_, which then drops to 4.9 throughout the experiment alongside a reduction in Euclidean distance (Figures 5 and 6). The trait, however, shows a sharp increase on Day 2 with the highest *ESPI recorded on day three at 26.7 and maintains a high *ESPI until Day 4. Fucoxanthin fluorescence increases in *g* from 3.59 to 6.15, while Euclidean distance drops from 1.73 to 1.4 from Days 1–7 (Figures 5, 6). *ESPI maintains relative stability across the experiment and peaks on Day 7. The variance of chlorophyll *a* content as measured by F680 remains moderate, starting at 1.15 and moving to 1.91. The Day 7 means are extremely high and variable; as such standardized effect size and *ESPI remain low (Figures 5 and 6). Day 4 again shows the greatest *ESPI. rETRmax reduces from Days 1 to 7 (2.75–1.75, respectively) (Figures 5 and 6), and Euclidean distance stays the same despite the conditions under which the variance was discovered being different. Day 3 presents the greatest *ESPI (at 10.3), after which values drop to the lowest plasticity out of the five and maintain stability until the end of the experiment.

## 4 Discussion

### 4.1 Simulation Dataset

The simulated dataset demonstrates the application of the proposed *ESPI and its statistical benefits in contrast to the original method. The dataset, built to represent a ‘worst case’ experimental outcome (Figure 2a–d), was used to contrast the statistical strength of both the original and newly proposed method.

While *ESPI exhibited an underestimation relative to ground truth (Figure 3b) as compared with original ESPI (Figure 3a), this error is consistent and systematic, irrespective of the number of environmental factors considered. Future study may be able to devise a correction factor or bias correction to address this and increase the reliability of the statistic. Bias and root mean squared error (RMSE) of *ESPI (Figure S1) decrease as the number of environmental factors considered increase, which is to be expected as once high dimensions are reached, as Euclidean distance can flatten at high dimensions. Figure 3b demonstrates this effect in which Euclidean distance calculated under higher dimensions ‘compresses’ *ESPI data, a consideration which must be taken when comparing across different experimental designs. One limitation of this method lies in that comparison of phenotypic plasticity when calculated using different numbers of environmental factors will result in different distributions of the data, with plasticity on average decreasing as environmental factors are added. This effect remains relatively small when low numbers of factors are considered (~5), however, as 10 factors are approached a greater change is seen. One potential solution in avoiding miscommunication of plasticity data may lie in reporting *ESPI to the number of dimensions used to calculate Euclidean distance, or in the substitution of Euclidean with Mahalonobis distance which more robustly accounts for correlation between factors (one of the causes of Euclidean distance compression). Euclidean distance is known to become less descriptive across all applications in something known as the ‘curse of dimensionality’, which describes Euclidean distance's ineffectiveness as dimensions increase (Xia et al. 2015). The number of these influences is not agreed upon, but they can be observed somewhere above 20 ‘factors’ or dimensions when modelled (Cabestany et al. 2005; Xia et al. 2015). This value is debated, and it is difficult to define a clear cut‐off point, however, this issue is unlikely to arise within the study of life sciences as experimental designs with this many variables would produce datasets of enormous complexity (Chitwood and Topp 2015; Houle et al. 2010; Tardieu et al. 2017; York 2019). Despite the error observed when comparing values to ground truth, *ESPI presents a much more stable measure of phenotype plasticity than ESPI as observed under a singular dimension, as seen through that markedly reduced CV (Figure 3c). The key advantages of *ESPI are however observable when considering axis and scale invariance (Figure 4a,b). ESPI is highly dependent on both environmental value, which is inherently only one dimensional. Furthermore, it cannot be applied for cross‐trait comparison as raw data scales are reflected in calculated values. Though *ESPI is dependent on Euclidean distance factor number, care must be taken when comparing any phenotypic data between studies due to the complex nature of phenotype expression (Chitwood and Topp 2015; Pieruschka and Schurr 2019), as such researchers must caveat cross‐study comparisons with the exact tested environmental variable number and type. Overall, *ESPI presents a robust and statistically conservative measure of phenotypic plasticity which is capable of handling multidimensional environmental gradients, and cross‐trait comparability.

### 4.2 *Thalassiosira weissfloggi* Phenotype Plasticity

Each trait observed in the case study of *T. weissflogii* exhibits patterns of plasticity over time, largely coherent with what is known of the physiology of said trait. Alpha, which defines the efficiency of photosynthesis under low light, exhibited a short‐term spike in plasticity under only modest environmental variation, peaking between Days 2 and 4, confirmed by wide confidence intervals and large differences in mean alpha performance even under the same irradiance (Figures 5 and 6). On Day 1, both the minimum and maximum performance were observed under low light, with the highest mean at the lower temperature. This may reflect a need to maintain a higher photosynthetic efficiency under low light in cooler temperatures to maintain performance when activity is lowered. Each of the highest means was discovered under a lowered irradiance respective to the pre‐experimental culture conditions, which was expected considering alpha's role in photosynthesis (Coles and Jones 2000; Laws and Mclellan 2020). By Day 7, alpha's *ESPI declined considerably, indicating this trait can demonstrate high plasticity under short‐term stress before alternative photosynthetic adaptation. It is worth noting that there is evidence suggesting that as cell size increases, alpha decreases in marine diatoms (Geider et al. 1986; Yan et al. 2018) and a separate investigation of prolonged responses regarding this relationship would be beneficial (Prins et al. 2020). Regulation of alpha early in the cell's growth cycle likely provides ecological benefits in that organisms are able to rapidly adapt to dynamic light fluctuations such as those provided by water column mixotrophy, or an algal bloom (Prins et al. 2020; Shatwell et al. 2012).

*E*_k_, the minimum saturating irradiance as determined by the maximum photosynthetic rate and alpha, maintained the highest *ESPI of all traits on Days 1 and 7, indicating strong and sustained phenotypic plasticity (Figures 5 and 6). Although *ESPI values decreased slightly from Days 1 to 7, *E*_k_ remained extremely sensitive to shifts in temperature and nutrient availability. The highest performance on both days was observed under higher temperatures, 12.8 μmol photons m^−2^ s^−1^ and 200% nutrient load, with a slightly elevated mean response on Day 1 as compared with Day 7. *E*_k_ also showed one of the most consistently high *ESPIs and followed a similar, though delayed, pattern to alpha, which may in part be that it is also governed by the P‐I curve (Lund‐Hansen et al. 2020; Petrou and Ralph 2011). *E*_k_ can be used as an indicator of photoadaptation, and the observed elevated *E*_k_ function at 12.8 μmol photons m^−2^ s^−1^ demonstrates the ability of *T. weissflogii* to increase light utilisation in low light conditions (Huovinen and Gómez 2011; Mock and Hoch 2005; Petrou and Ralph 2011). Wide variation in mean under this low light condition is positively correlated with an increase in temperature and nutrient load, indicating these factors to be performance limiters. Similar to alpha, plasticity in the minimum saturating efficiency of diatoms likely provides benefits in early adjustment to variable conditions. It is known that various algae can downregulate *E*_k_ in response to low light, so that the minimum energy load is higher than what is required for growth (Gómez et al. 2009). A decrease in respiration is a common strategy for polar diatoms which survive up to 5 months in darkness (Gómez et al. 2009; Kühl et al. 2001).

For rETRmax, Day 1 responses displayed a pronounced phenotypic shift despite relatively small Euclidean distance, suggesting a strong immediate reaction to elevated temperature and irradiance. By Day 7, rETRmax performance still peaked but with a lower *ESPI, indicating a level of acclimation to these conditions. This pattern is also consistent with the possibility of a photosynthetic threshold (photoinhibition) at higher irradiance intensities (Ihnken et al. 2010; Perkins et al. 2006), above which rETRmax gains are limited. This trait shows some of the lowest plasticity except for on Day 3, where a spike occurs, produced by a high Hedges' *g* and moderate Euclidean distance (Figures 5 and 6). Previous work has shown that rETRmax shows a similar pattern of variation based on temperature across eight marine diatoms, which may relate to its being a central function of photosynthesis (Claquin et al. 2008). It may be plausible that plasticity is inherently lowered in traits with key roles in cellular function or ones which are a cumulation of many cellular mechanisms coming together. When rETRmax is elevated, diatoms may be capable of rapid growth under variable light conditions or high light (Gilbert et al. 2000; Wilhelm and Jakob 2011), however, maintaining elevated rETRmax and photosynthesis in general incurs metabolic costs (Li et al. 2015; Litchman et al. 2007). Thus, down‐regulating rETRmax to track ambient light levels may be the more stable strategy.

Fucoxanthin content exhibited a wide variation on Day 1, positively correlated with temperature and nutrient load, and negatively with irradiance. Ecologically, fucoxanthin and other accessory pigments function to regulate light capture (Prins et al. 2020; Wulff et al. 2005), and these environmental shifts may have stimulated photosynthetic activity and supported upregulated pigment production. High nutrient load has previously been shown to increase fucoxanthin content in the species (Marella and Tiwari 2020; Supramaetakorn et al. 2019), and other diatom species are reported to have the highest dry weight when cultured around 25°C (Khaw et al. 2022). By Day 7 (Figures 5 and 6), the highest mean values were observed under lower light (25 μmol photons m^−2^ s^−1^) and higher temperature, albeit with reduced nutrient levels compared with Day 1. The delay in reaching these peak pigment values suggests that pigment accumulation is not an immediate response but requires more adaptation time. Interestingly, mean fucoxanthin content declined across the two time points; however, it still yielded a higher *ESPI at Day 7, which suggests partial acclimation or resource reallocation throughout the experiment (Prins et al. 2020; Wulff et al. 2005).

In contrast, chlorophyll *a* content consistently showed a smaller *ESPI and narrower confidence intervals, indicating less variance among the two groups (Figures 5 and 6). The trait's role in cellular metabolism appears to constrain its plasticity, thus limiting the extent of its response under the tested environmental gradients. The stability of chlorophyll *a* may also be reflective of an energy conservation strategy, in that it is kept consistent to maintain energy capture under short‐term environmental fluctuations. As the measure of chlorophyll *a* in this case study was only through spectroscopy, it is hard to detangle whether the observed result comes from a variation in chlorophyll *a* content per cell or if this is the result of the aggregated biomass increase. The pattern in the *ESPI observed does, however, point to the highest plasticity occurring on Day 4, as with many other traits observed here.

The *ESPI results underscore the complex interplay of trait plasticity under multi‐factor environmental drivers. Some traits (*E*_k_, Fucoxanthin) show persistent or enhanced divergence over time, whereas others (alpha, rETRmax) exhibit an early spike in plasticity followed by partial acclimation as cultures adjust to new environmental conditions. Traits where elevated functionality incurs considerable metabolic costs display trade‐offs between nutrient allocation and photosynthetic regulation, dictated by the advantage of timely adjustment. There is a clear difference in plastic response between fast‐acting regulatory mechanisms versus those that cannot be immediately altered by the cell or have certain core functions; however, each trait displays a unique pattern, with different distribution patterns across these spaces.

Days 3 and 4 post‐inoculation under a new set of environmental factors presents a window of elevated plasticity in this marine diatom, with the highest plasticity seen across four of the traits on these 2 days. Previously, growth rate has been determined as a ‘master trait’ that guides and has complex trade‐offs with acclamatory behaviours (Bastos 2022; Klausmeier et al. 2004; Litchman et al. 2007). Under variation in environmental conditions, a trade‐off between growth and competitive adaptation allows for different species to co‐exist; however, marine diatoms have been shown to take a ‘velocity strategy’ which focuses on rapid nutrient uptake and high maximum growth rate (Crowlfiy 1975; Litchman et al. 2007; Sommer 1984). The exponential growth phase was found to correlate with highest plasticity for all conditions under which they were found (Figure 6, Figure S2). This leads to the conclusion that diatoms may undergo a period of adjustment during the initial exponential phase in which their plasticity allows them to adapt to the present environmental conditions. This finding may be a result of their ability for rapid nutrient uptake and photoacclimation, a strategy allowing for rapid phenotypic plasticity under changing environmental factors, giving the population an opportunity to determine and find the most appropriate adaptations (Ajani et al. 2021; Litchman et al. 2007; Peirson 2015). This possible response is likely a conglomeration of many cellular processes that are elevated under exponential phase, and an abundance of nutrients at this period in the experiment. The diatom *Skeletonema marinoi* was shown to produce the highest quantity of various metabolites specifically under exponential phase and was assumed to have highest overall metabolism during this period (Vidoudez and Pohnert 2012). Furthermore, variation is often regulated by intracellular metabolism (leading to downstream epigenetic modulation) and could act to influence epigenetic modulation which is likely to play a role in observed plasticity (Kaelin and McKnight 2013; Vidoudez and Pohnert 2012). Though the exact mechanisms which influence plasticity are debated and poorly understood on a molecular level, inherently, under exponential phase, the culture near ubiquitously undergoes the highest rate of cell division. The process of rapid asexual, and perhaps sexual, reproduction of the diatom in this phase of growth suggests that there is a higher rate of differentiation across the population. Perhaps the observation of higher plasticity at early exponential phase can be attributed to the increased diversity seen across daughter cells before selective pressures which allow for the dominance of a most appropriate ‘mono‐phenotype.’ This model would align closely with the theory that plasticity behaves as a mechanism that allows for evolution to occur, by producing variations across a culture enough to keep a viable population, after which suitable adaptations are selected for (Ashe et al. 2021; Duncan et al. 2014).

How this interplays within an entire population remains to be understood, as considering the genetic diversity of diatoms, the likelihood of uniform phenotypic plasticity across a culture would seem unlikely. Further studies comparing the differing responses of various algal families may shed further light on how these mechanisms behave. This case study is also limited in that pure lab‐grown cultures vary greatly from natural communities of diatoms. Wild‐type diatom assemblages will be influenced by microbial populations, predation, and competition, all of which cannot be accounted for in this dataset which was observed under pure culture (Morin et al. 2015; Neury‐Ormanni et al. 2020; Sjöqvist and Kremp 2016), a common issue seen across *ex situ* work. The biological triggers which become active under these various pressures likely result in a breadth of plasticity which is not observed here. Investigations which account for natural community structures or those which utilize more recently isolated diatoms may be able to more comprehensively address the questions of plasticity raised in this case study.

The findings also highlight a need for targeted epigenetics studies which investigate differences throughout different periods of growth, and how these link to observed plasticity. This may help validate whether phenotypic plasticity undergoes windows of elevation, considering epigenetics changes are highly likely to be the primary mechanism behind plasticity (Feinberg 2007; Huang et al. 2019; Schlichting and Wund 2014).

## 5 Conclusion

The methodology presented here provides an adapted option for calculating phenome plasticity in biology. The proposed *ESPI utilising a measure of variance and Euclidean distance aims to allow for a flexible method of plasticity quantification that is straightforward to apply and digest as a reader. The case study of *T. weissflogii* demonstrates that plasticity in this marine diatom is temporal and closely linked to the growth stage, with the early exponential phase displaying elevated plasticity in traits that act to adapt the culture to new environmental conditions quickly. The statistical method described aims to increase the accessibility of phenome quantification to enhance our understanding of adaptability, population dynamics, and how crucial organisms may respond to changing environments.

## Author Contributions

**Lilian Hoch:** conceptualization (equal), data curation (lead), formal analysis (equal), investigation (lead), methodology (equal), visualization (equal), writing – original draft (lead), writing – review and editing (equal). **Andrei Herdean:** conceptualization (equal), data curation (equal), formal analysis (equal), investigation (equal), methodology (equal), resources (equal), supervision (equal), visualization (equal), writing – original draft (equal), writing – review and editing (equal). **Stephen Woodcock:** validation (equal), writing – review and editing (equal). **Kittikun Songsomboon:** software (equal), validation (equal), writing – review and editing (equal). **Breanna Osborne:** writing – review and editing (supporting). **Peter J. Ralph:** conceptualization (equal), resources (equal), supervision (lead), writing – review and editing (equal).

## Funding

The authors have nothing to report.

## Conflicts of Interest

The authors declare no conflicts of interest.

## Supporting information


**Figure S1:** Bias and RMSE of each *ESPI and original ESPI. Bias and RMSE are calculated using ground truth determined through the simulation dataset (a). Bias of each *ESPI and ESPI when calculated under 1 environmental dimension with CIs (95%). (b) RMSE of each *ESPI and ESPI when calculated under 1 environmental dimension with CIs (95%). (c) Bias of *ESPI when calculated under 2, 4, 5, and 10 environmental gradients with CIs (95%). (d) RMSE of *ESPI when calculated under 2, 4, 5, and 10 environmental gradients with CIs (95%).
**Figure S2:** Histogram of OD720 of *Thalassiosira weissflogii* culture per environmental condition, as a proxy for biomass accumulation with standard error. Environmental conditions are indicated in the top left of each panel. Panels A–H are separated by temperature and light combinations with two temperatures (20°C and 24°C) and four irradiance levels under each (13, 25, 57, 166 μmol photons m^−2^ s^−1^). Colour is indicative of the nutrient concentration under each environmental condition (100%, 50%, and 200%). Daily data is constructed from the average OD720 per day of 192 replicates with error bars representing the standard deviation of each group.
**Table S1:** Conditions matrix demonstrating the range of abiotic conditions *T. weissfloggi* was grown for 7 days. All light and nutrient conditions were repeated in each 20°C and 24°C. Nutrient concentration refers to the concertation of F/2 medium, which has halved and doubled, respectively.
**Equation S1**. Calculation of correction factor *J*(*v*) for the transformation of Cohens' *d* to Hedge's *g*. *v* represents degrees of freedom, and Γ the gamma function.

## Data Availability

The data that support the findings of this study are openly availiable in Dryad at DOI: https://doi.org/10.5061/dryad.cfxpnvxmz.

## References

[ece373072-bib-0001] Ajani, P. A. , K. Petrou , M. E. Larsson , D. A. Nielsen , J. Burke , and S. A. Murray . 2021. “Phenotypic Trait Variability as an Indication of Adaptive Capacity in a Cosmopolitan Marine Diatom.” Environmental Microbiology 23, no. 1: 207–223. 10.1111/1462-2920.15294. 33118307

[ece373072-bib-0002] Alverson, A. J. , B. Beszteri , M. L. Julius , and E. C. Theriot . 2011. “The Model Marine Diatom *Thalassiosira pseudonana* Likely Descended From a Freshwater Ancestor in the Genus Cyclotella.” BMC Evolutionary Biology 11, no. 1: 125. 10.1186/1471-2148-11-125. 21569560 PMC3121624

[ece373072-bib-0003] Argyle, P. A. , J. Hinners , N. G. Walworth , S. Collins , N. M. Levine , and M. A. Doblin . 2021. “A High‐Throughput Assay for Quantifying Phenotypic Traits of Microalgae.” Frontiers in Microbiology 12. 10.3389/fmicb.2021.706235. PMC8528002 34690950

[ece373072-bib-0004] Ashe, A. , V. Colot , and B. P. Oldroyd . 2021. “How Does Epigenetics Influence the Course of Evolution?” Philosophical Transactions of the Royal Society B 376, no. 1826. 10.1098/rstb.2020.0111. PMC8059608 33866814

[ece373072-bib-0005] Bastos, A. O. 2022. Phenotypic Plasticity in Benthic Diatoms: Cellular Motility and Photoacclimation Capacity. University of Aveiro.

[ece373072-bib-0006] Beier, S. , A. R. Rivers , M. A. Moran , and I. Obernosterer . 2015. “Phenotypic Plasticity in Heterotrophic Marine Microbial Communities in Continuous Cultures.” ISME Journal 9, no. 5: 1141–1151. 10.1038/ismej.2014.206. 25397947 PMC4409158

[ece373072-bib-0007] Cabestany, J. , A. Prieto , D. F. Sandoval , M. Verleysen , and D. François . 2005. “The Curse of Dimensionality in Data Mining and Time Series Prediction.” In LNCS (Vol. 3512). www.ucl.ac.be/mlg.

[ece373072-bib-0008] Champely, S. , and D. Chessel . 2002. “Measuring Biological Diversity Using Euclidean Metrics.” Environmental and Ecological Statistics 9, no. 2: 167–177. 10.1023/A:1015170104476.

[ece373072-bib-0009] Cheng, L. , J. Abraham , K. E. Trenberth , et al. 2023. “Another Year of Record Heat for the Oceans.” Advances in Atmospheric Sciences 40, no. 6: 963–974. 10.1007/s00376-023-2385-2. 36643611 PMC9832248

[ece373072-bib-0010] Cheng, L. , J. Abraham , K. E. Trenberth , et al. 2025. “Record High Temperatures in the Ocean in 2024.” Advances in Atmospheric Sciences 42, no. 6: 1092–1109. 10.1007/s00376-025-4541-3.

[ece373072-bib-0011] Chitwood, D. H. , and C. N. Topp . 2015. “Revealing Plant Cryptotypes: Defining Meaningful Phenotypes Among Infinite Traits.” Current Opinion in Plant Biology 24: 54–60. 10.1016/j.pbi.2015.01.009. 25658908

[ece373072-bib-0012] Claquin, P. , I. Probert , S. Lefebvre , and B. Veron . 2008. “Effects of Temperature on Photosynthetic Parameters and TEP Production in Eight Species of Marine Microalgae.” Aquatic Microbial Ecology 51, no. 1: 1–11. 10.3354/ame01187.

[ece373072-bib-0013] Cohen, J. 1969. Statistical Power Analysis for the Behavioral Sciences. 2nd ed. Lawrence Erlbaum Associates, Publishers.

[ece373072-bib-0014] Cohen, J. 1992. “Quantitative Methods in Psychology: A Power Primer.” Psychological Bulletin 112, no. 1: 155–159. 19565683 10.1037//0033-2909.112.1.155

[ece373072-bib-0015] Coles, J. F. , and R. C. Jones . 2000. “Effect of Temperature on Photosynthesis‐Light Response and Growth of Four Phytoplankton Species Isolated From a Tidal Freshwater River.” Journal of ‐Phycology 36: 7–16.

[ece373072-bib-0016] Crowlfiy, P. H. 1975. “Natural Selection and the Michaelis Constant.” Journal of Theoretical Biology 50: 461–475. 1134108 10.1016/0022-5193(75)90093-4

[ece373072-bib-0017] Duncan, E. J. , P. D. Gluckman , and P. K. Dearden . 2014. “Epigenetics, Plasticity, and Evolution: How Do We Link Epigenetic Change to Phenotype?” Journal of Experimental Zoology Part B: Molecular and Developmental Evolution 322, no. 4: 208–220. 10.1002/jez.b.22571. 24719220

[ece373072-bib-0018] Falkowski, P. , R. J. Scholes , E. Boyle , et al. 2000. “The Global Carbon Cycle: A Test of Our Knowledge of Earth as a System.” Science 290, no. 5490: 291–296. 10.1126/science.290.5490.291. 11030643

[ece373072-bib-0019] Feinberg, A. P. 2007. “Phenotypic Plasticity and the Epigenetics of Human Disease.” Nature 447, no. 7143: 433–440. 10.1038/nature05919. 17522677

[ece373072-bib-0020] Field, C. B. , M. J. Behrenfeld , J. T. Randerson , and P. Falkowski . 1976. “Primary Production of the Biosphere.” Biochemical Society Transactions 4, no. 5: 954. 10.1042/bst0040954.

[ece373072-bib-0021] Furbank, R. T. , and M. Tester . 2011. “Phenomics—Technologies to Relieve the Phenotyping Bottleneck.” Trends in Plant Science 16, no. 12: 635–644. 10.1016/j.tplants.2011.09.005. 22074787

[ece373072-bib-0022] García, N. , J. A. López‐Elías , A. Miranda , M. Martínez‐Porchas , N. Huerta , and A. García . 2012. “Effect of Salinity on Growth and Chemical Composition of the Diatom *Thalassiosira weissflogii* at Three Culture Phases.” Latin American Journal of Aquatic Research 40, no. 2: 435–440. 10.3856/vol40-issue2-fulltext-18.

[ece373072-bib-0023] Geider, R. , T. Piatt , and J. Raven . 1986. “Size Dependence of Growth and Photosynthesis in Diatoms: A Synthesis.” Marine Ecology Progress Series 30: 93–104. 10.3354/meps030093.

[ece373072-bib-0024] Gilbert, M. , A. Domin , A. Becker , and C. Wilhelm . 2000. “Estimation of Primary Productivity by Chlorophyll a In Vivo Fluorescence in Freshwater Phytoplankton.” Photosynthetica 38: 111–126.

[ece373072-bib-0025] Gómez, I. , A. Wulff , M. Y. Roleda , et al. 2009. “Light and Temperature Demands of Marine Benthic Microalgae and Seaweeds in Polar Regions.” Botanica Marina 52, no. 6: 593–608. 10.1515/BOT.2009.073.

[ece373072-bib-0026] Goulet‐Pelletier, J.‐C. , and D. Cousineau . 2018. “A Review of Effect Sizes and Their Confidence Intervals, Part I: The Cohen's d Family.” Quantitative Methods for Psychology 14, no. 4: 242–265. 10.20982/tqmp.14.4.p242.

[ece373072-bib-0027] Guillard, R. R. L. , and J. H. Ryther . 1962. “Studies of Marine Planktonic Diatoms. I. Cyclotella Nana Hustedt, and *Detonula confervacea* (CLEVE).” Canadian Journal of Microbiology 8, no. 1140: 229–239. 13902807 10.1139/m62-029

[ece373072-bib-0028] Harrison, W. G. , and G. F. Cota . 1991. “Primary Production in Polar Waters: Relation to Nutrient Availability.” Polar Research 10, no. 1: 87–104. 10.3402/polar.v10i1.6730.

[ece373072-bib-0029] Hedges, L. V. 1981. “Distribution Theory for Glass's Estimator of Effect Size and Related Estimators.” Journal of Educational Statistics 6, no. 2: 107–128.

[ece373072-bib-0030] Houle, D. , D. R. Govindaraju , and S. Omholt . 2010. “Phenomics: The Next Challenge.” Nature Reviews Genetics 11, no. 12: 855–866. 10.1038/nrg2897. 21085204

[ece373072-bib-0031] Huang, R. , J. Ding , K. Gao , et al. 2019. “A Potential Role for Epigenetic Processes in the Acclimation Response to Elevated pco_2_ in the Model Diatom *Phaeodactylum tricornutum* .” Frontiers in Microbiology 10. 10.3389/fmicb.2018.03342. PMC6376209 30800105

[ece373072-bib-0032] Huovinen, P. , and I. Gómez . 2011. “Spectral Attenuation of Solar Radiation in Patagonian Fjord and Coastal Waters and Implications for Algal Photobiology.” Continental Shelf Research 31, no. 3–4: 254–259. 10.1016/j.csr.2010.09.004.

[ece373072-bib-0033] Ihnken, S. , A. Eggert , and J. Beardall . 2010. “Exposure Times in Rapid Light Curves Affect Photosynthetic Parameters in Algae.” Aquatic Botany 93, no. 3: 185–194. 10.1016/j.aquabot.2010.07.002.

[ece373072-bib-0034] Ji, X. , J. M. H. Verspagen , D. B. Van De Waal , B. Rost , and J. Huisman . 2020. “Phenotypic Plasticity of Carbon Fixation Stimulates Cyanobacterial Blooms at Elevated CO_2_ .” Science Advances 6: eaax2926. 10.1126/sciadv.aax2926 PMC7030920 32128392

[ece373072-bib-0035] Jiang, L. Q. , A. Kozyr , J. M. Relph , et al. 2023. “The Ocean Carbon and Acidification Data System.” Scientific Data 10, no. 1: 1–12. 10.1038/s41597-023-02042-0. 36922515 PMC10017681

[ece373072-bib-0036] Kaelin, W. G. , and S. L. McKnight . 2013. “Influence of Metabolism on Epigenetics and Disease.” Cell 153, no. 1: 56–69. 10.1016/j.cell.2013.03.004. 23540690 PMC3775362

[ece373072-bib-0037] Khaw, Y. S. , F. M. Yusoff , H. T. Tan , et al. 2022. “Fucoxanthin Production of Microalgae Under Different Culture Factors: A Systematic Review.” Marine Drugs 20, no. 10. 10.3390/md20100592. PMC9604996 36286416

[ece373072-bib-0038] Kietpawpan, M. , P. Visuthismajarn , and C. Ratanachai . 2003. “Statistical Assessment of Trophic Conditions: Squared Euclidean Distance Approach.” Songklanakarin Journal of Science and Technology 25, no. 3: 359–365.

[ece373072-bib-0039] Klausmeier, C. A. , E. Litchman , and S. A. Levin . 2004. “Phytoplankton Growth and Stoichiometry Under Multiple Nutrient Limitation.” Limnology and Oceanography 49, no. 4 II: 1463–1470. 10.4319/lo.2004.49.4_part_2.1463.

[ece373072-bib-0040] Kühl, M. , R. N. Glud , J. Borum , R. Roberts , and S. Rysgaard . 2001. “Photosynthetic Performance of Surface‐Associated Algae Below Sea Ice as Measured With a Pulse‐Amplitude‐Modulated (PAM) Fluorometer and O2 Microsensors.” Marine Ecology Progress Series 223: 1–14.

[ece373072-bib-0041] Laitinen, R. A. E. , and Z. Nikoloski . 2019. “Genetic Basis of Plasticity in Plants.” Journal of Experimental Botany 70, no. 3: 795–804. 10.1093/jxb/ery404. 30445526

[ece373072-bib-0042] Laws, E. , and A. Mclellan . 2020. “Interactive Effects of CO_2_, Temperature, Irradiance and Nutrient Limitation of the Growth and Physiology of the Marine Diatom *Thalassiosira pseudonana* (Coscinodiscophyceae).” 10.1111/jpy.13048 32750165

[ece373072-bib-0043] Leung, C. , M. Rescan , D. Grulois , and L. M. Chevin . 2020. “Reduced Phenotypic Plasticity Evolves in Less Predictable Environments.” Ecology Letters 23, no. 11: 1664–1672. 10.1111/ele.13598. 32869431 PMC7754491

[ece373072-bib-0044] Li, G. , C. M. Brown , J. A. Jeans , N. A. Donaher , A. Mccarthy , and D. A. Campbell . 2015. “The Nitrogen Costs of Photosynthesis in a Diatom Under Current and Future pCO2.” New Phytologist 205, no. 2: 533–543. 10.1111/nph.13037. 25256155

[ece373072-bib-0045] Lin, W. J. , H. C. Ho , S. C. Chu , and J. Y. Chou . 2020. “Effects of Auxin Derivatives on Phenotypic Plasticity and Stress Tolerance in Five Species of the Green Alga Desmodesmus (Chlorophyceae, Chlorophyta).” PeerJ 2020, no. 3. 10.7717/peerj.8623. PMC7067201 32195045

[ece373072-bib-0046] Litchman, E. , C. A. Klausmeier , O. M. Schofield , and P. G. Falkowski . 2007. “The Role of Functional Traits and Trade‐Offs in Structuring Phytoplankton Communities: Scaling From Cellular to Ecosystem Level.” Ecology Letters 10, no. 12: 1170–1181. 10.1111/j.1461-0248.2007.01117.x. 17927770

[ece373072-bib-0047] Lund‐Hansen, L. C. , I. Hawes , K. Hancke , et al. 2020. “Effects of Increased Irradiance on Biomass, Photobiology, Nutritional Quality, and Pigment Composition of Arctic Sea Ice Algae.” Marine Ecology Progress Series 648: 95–110. 10.3354/meps13411.

[ece373072-bib-0048] Marella, T. K. , and A. Tiwari . 2020. “Marine Diatom *Thalassiosira weissflogii* Based Biorefinery for Co‐Production of Eicosapentaenoic Acid and Fucoxanthin.” Bioresource Technology 307: 123245. 10.1016/j.biortech.2020.123245. 32234591

[ece373072-bib-0049] Marinov, I. , S. C. Doney , and I. D. Lima . 2010. “Response of Ocean Phytoplankton Community Structure to Climate Change Over the 21st Century: Partitioning the Effects of Nutrients, Temperature and Light.” Biogeosciences 7, no. 12: 3941–3959. 10.5194/bg-7-3941-2010.

[ece373072-bib-0050] Mock, T. , and N. Hoch . 2005. “Long‐Term Temperature Acclimation of Photosynthesis in Steady‐State Cultures of the Polar Diatom *Fragilariopsis cylindrus* .” Photosynthesis Research 85: 307–317. 16170633 10.1007/s11120-005-5668-9

[ece373072-bib-0077] Morales, E. A. , and F. R. Trainor . 1997. “Algal Phenotypic Plasticity: Its Importance in Developing New Concepts The Case for *Scenedesmus* .” Algae (The Korean Journal of Phycology) 12, no. 3: 147–157.

[ece373072-bib-0051] Morin, S. , B. Bonet , N. Corcoll , H. Guasch , M. Bottin , and M. Coste . 2015. “Cumulative Stressors Trigger Increased Vulnerability of Diatom Communities to Additional Disturbances.” Microbial Ecology 70, no. 3. 10.1007/s00248-015-0602-y. 25896427

[ece373072-bib-0052] Neury‐Ormanni, J. , J. Vedrenne , and S. Morin . 2020. “Benthic Diatom Growth Kinetics Under Combined Pressures of Microalgal Competition, Predation and Chemical Stressors.” Science of the Total Environment 734: 139484. 32464387 10.1016/j.scitotenv.2020.139484

[ece373072-bib-0053] Nicotra, A. B. , O. K. Atkin , S. P. Bonser , et al. 2010. “Plant Phenotypic Plasticity in a Changing Climate.” Trends in Plant Science 15, no. 12: 684–692. 20970368 10.1016/j.tplants.2010.09.008

[ece373072-bib-0054] Peirson, B. R. E. 2015. “Plasticity, Stability, and Yield: The Origins of Anthony David Bradshaw's Model of Adaptive Phenotypic Plasticity.” Studies in History and Philosophy of Science Part C: Studies in History and Philosophy of Biological and Biomedical Sciences 50: 51–66. 10.1016/j.shpsc.2015.01.005. 25641217

[ece373072-bib-0055] Pélabon, C. , C. H. Hilde , S. Einum , and M. Gamelon . 2020. “On the Use of the Coefficient of Variation to Quantify and Compare Trait Variation.” Evolution Letters 4, no. 3: 180–188. 10.1002/evl3.171. 32547779 PMC7293077

[ece373072-bib-0056] Perkins, R. G. , J. L. Mouget , S. Lefebvre , and J. Lavaud . 2006. “Light Response Curve Methodology and Possible Implications in the Application of Chlorophyll Fluorescence to Benthic Diatoms.” Marine Biology 149, no. 4: 703–712. 10.1007/s00227-005-0222-z.

[ece373072-bib-0057] Petrou, K. , and P. J. Ralph . 2011. “Photosynthesis and Net Primary Productivity in Three Antarctic Diatoms: Possible Significance for Their Distribution in the Antarctic Marine Ecosystem.” Marine Ecology Progress Series 437: 27–40. 10.3354/meps09291.

[ece373072-bib-0079] Pieruschka, R. , and U. Schurr . 2019. “Plant Phenotyping: Past, Present, and Future.” Plant Phenomics 2019: 1–6. 10.34133/2019/7507131. PMC7718630 33313536

[ece373072-bib-0058] Prins, A. , P. Deleris , C. Hubas , and B. Jesus . 2020. “Effect of Light Intensity and Light Quality on Diatom Behavioral and Physiological Photoprotection.” Frontiers in Marine Science 7. 10.3389/fmars.2020.00203.

[ece373072-bib-0059] Sackett, O. , K. Petrou , B. Reedy , et al. 2013. “Phenotypic Plasticity of Southern Ocean Diatoms: Key to Success in the Sea Ice Habitat?” PLoS One 8, no. 11: 1–12. 10.1371/journal.pone.0081185. PMC3868450 24363795

[ece373072-bib-0060] Schlichting, C. D. , and M. A. Wund . 2014. “Phenotypic Plasticity and Epigenetic Marking: An Assessment of Evidence for Genetic Accommodation.” Evolution 68, no. 3: 656–672. 10.1111/evo.12348. 24410266

[ece373072-bib-0061] Shatwell, T. , A. Nicklisch , and J. Köhler . 2012. “Temperature and Photoperiod Effects on Phytoplankton Growing Under Simulated Mixed Layer Light Fluctuations.” Limnology and Oceanography 57, no. 2: 541–553. 10.4319/lo.2012.57.2.0541.

[ece373072-bib-0062] Sjöqvist, C. O. , and A. Kremp . 2016. “Genetic Diversity Affects Ecological Performance and Stress Response of Marine Diatom Populations.” ISME Journal 10, no. 11: 2755–2766. 10.1038/ismej.2016.44. 27046335 PMC5113836

[ece373072-bib-0063] Sommer, U. 1984. “The Paradox of the Plankton: Fluctuations of Phosphorus Availability Maintain Diversity of Phytoplankton in Flow‐Through Cultures.” Limnology and Oceanography 29, no. 3: 633–636. 10.4319/lo.1984.29.3.0633.

[ece373072-bib-0064] Stomp, M. , M. A. Van Dijk , H. M. J. Van Overzee , et al. 2008. “The Timescale of Phenotypic Plasticity and Its Impact on Competition in Fluctuating Environments.” American Naturalist 172, no. 5. 10.1086/591680. 18828745

[ece373072-bib-0065] Supramaetakorn, W. , S. Meksumpun , K. Ichimi , N. Thawonsode , and O. Veschasit . 2019. “Potential Fucoxanthin Production From a Marine Diatom.” Journal of Fisheries and Environment 43, no. 3: 1–10.

[ece373072-bib-0066] Tardieu, F. , L. Cabrera‐Bosquet , T. Pridmore , and M. Bennett . 2017. “Plant Phenomics, From Sensors to Knowledge.” Current Biology 27, no. 15: R770–R783. 10.1016/j.cub.2017.05.055. 28787611

[ece373072-bib-0067] Valladares, F. , D. Sanchez‐Gomez , and M. A. Zavala . 2006. “Quantitative Estimation of Phenotypic Plasticity: Bridging the Gap Between the Evolutionary Concept and Its Ecological Applications.” Journal of Ecology 94, no. 6: 1103–1116. 10.1111/j.1365-2745.2006.01176.x.

[ece373072-bib-0068] Vidoudez, C. , and G. Pohnert . 2012. “Comparative Metabolomics of the Diatom Skeletonema Marinoi in Different Growth Phases.” Metabolomics 8, no. 4: 654–669. 10.1007/s11306-011-0356-6.

[ece373072-bib-0069] Wasserman, S. , L. V. Hedges , and I. Olkin . 1988. “Statistical Methods for Meta‐Analysis.” Journal of Educational Statistics 13, no. 1: 75. 10.2307/1164953.

[ece373072-bib-0070] West‐Eberhard, M. J. 2005. “Developmental Plasticity and the Origin of Species Differences.” Proceedings of the National Academy of Sciences of the United States of America 102, no. 1: 6543–6549. 10.1073/pnas.0501844102. 15851679 PMC1131862

[ece373072-bib-0071] Wilhelm, C. , and T. Jakob . 2011. “From Photons to Biomass and Biofuels: Evaluation of Different Strategies for the Improvement of Algal Biotechnology Based on Comparative Energy Balances.” Applied Microbiology and Biotechnology 92, no. 5: 909–919. 10.1007/s00253-011-3627-2. 22005740

[ece373072-bib-0072] Wulff, A. , S. Vilbaste , and J. Truu . 2005. “Depth Distribution of Photosynthetic Pigments and Diatoms in the Sediments of a Microtidal Fjord.” Hydrobiologia 534, no. 1–3: 117–130. 10.1007/s10750-004-1417-x.

[ece373072-bib-0073] Xia, S. , Z. Xiong , Y. Luo , W. Xu , and G. Zhang . 2015. “Effectiveness of the Euclidean Distance in High Dimensional Spaces.” Optik 126, no. 24: 5614–5619. 10.1016/j.ijleo.2015.09.093.

[ece373072-bib-0074] Yan, D. , J. Beardall , and K. Gao . 2018. “Variation in Cell Size of the Diatom *Coscinodiscus granii* Influences Photosynthetic Performance and Growth.” Photosynthesis Research 137, no. 1: 41–52. 10.1007/s11120-017-0476-6. 29322482

[ece373072-bib-0075] Yang, D. , Y. Jiang , and F. He . 2009. “An Integrated View of the Correlations Between Genomic and Phenomic Variables.” Journal of Genetics and Genomics 36, no. 11: 645–651. 10.1016/S1673-8527(08)60156-3. 19932460

[ece373072-bib-0076] York, L. M. 2019. “Functional Phenomics: An Emerging Field Integrating High‐Throughput Phenotyping, Physiology, and Bioinformatics.” Journal of Experimental Botany 70, no. 2: 379–386. 10.1093/jxb/ery379. 30380107

