# Supplementary material for: New Method for Quantification of Phenotypic Plasticity Reveals How Plasticity Changes Over Time in the Diatom Thalassiosira weissflogii
Source: Ecol Evol. 2026 Feb 17;16(2):e73072. doi: 10.1002/ece3.73072 (PMC12912934; doi:10.1002/ece3.73072)
Supplement: Supplementary file 1 — Figure S1: Bias and RMSE of each *ESPI and original ESPI. Bias and RMSE are calculated using ground truth determined through the simulation dataset (a). Bias of each *ESPI and ESPI when calculated under 1 environmental dimension with CIs (95%). (b) RMSE of each *ESPI and ESPI when calculated under 1 environmental dimension with CIs (95%). (c) Bias of *ESPI when calculated under 2, 4, 5, and 10 environmental gradients with CIs (95%). (d) RMSE of *ESPI when calculated under 2, 4, 5, and 10 environmental gradients with CIs (95%). Figure S2: Histogram of OD720 of Thalassiosira weissflogii culture per environmental condition, as a proxy for biomass accumulation with standard error. Environmental conditions are indicated in the top left of each panel. Panels A–H are separated by temperature and light combinations with two temperatures (20°C and 24°C) and four irradiance levels under each (13, 25, 57, 166 μmol photons m−2 s−1). Colour is indicative of the nutrient concentration under each environmental condition (100%, 50%, and 200%). Daily data is constructed from the average OD720 per day of 192 replicates with error bars representing the standard deviation of each group. Table S1: Conditions matrix demonstrating the range of abiotic conditions T. weissfloggi was grown for 7 days. All light and nutrient conditions were repeated in each 20°C and 24°C. Nutrient concentration refers to the concertation of F/2 medium, which has halved and doubled, respectively. Equation S1. Calculation of correction factor J(v) for the transformation of Cohens' d to Hedge's g. v represents degrees of freedom, and Γ the gamma function. [file ECE3-16-e73072-s001.docx]

**Supplementary**

(Supp Eq. 1) $J(v)=\frac{\Gamma(\frac{1}{2}v)}{\sqrt{\frac{v}{2}\Gamma(\frac{1}{2}(v}-1))}$

**Supplementary Equation 1.** Calculation of correction factor *J(v)* for the transformation of Cohens’ *d* to Hedge’s *g*. *v* represents degrees of freedom, and $\Gamma$ the gamma function.

**

**

**Supplementary Figure 1.** Bias and RMSE of each *ESPI and original ESPI. Bias and RMSE are calculated using ground truth determined through the simulation dataset **1a.** Bias of each *ESPI and ESPI when calculated under 1 environmental dimension with CIs (95%). **1b**. RMSE of each *ESPI and ESPI when calculated under 1 environmental dimensional with CIs (95%). **1c.** Bias of *ESPI when calculated under 2, 4, 5 and 10 environmental gradients with CIs (95%). **1d.** RMSE of *ESPI when calculated under 2, 4, 5 and 10 environmental gradients with CIs (95%).

**Supplementary Table 1.** Conditions matrix demonstrating the range of abiotic conditions *T. weissfloggi* was grown under for 7 days. All light and nutrient conditions were repeated in each 20 °C and 24 °C. Nutrient concertation refers to the concertation of F/2 medium, which has halved and doubled respectively.

| **Temperature** | **Irradiance** | **Nutrient Concentration** |
| --- | --- | --- |
| 20 °C | 13 µmol photons m^-2^ s^-1^ | 50% |
|  |  | 100% |
|  |  | 200% |
|  | 25 µmol photons m^-2^ s^-1^ | 50% |
|  |  | 100% |
|  |  | 200% |
|  | 57 µmol photons m^-2^ s^-1^ | 50% |
|  |  | 100% |
|  |  | 200% |
|  | 166 µmol photons m^-2^ s^-1^ | 50% |
|  |  | 100% |
|  |  | 200% |
| 24 °C | 13 µmol photons m^-2^ s^-1^ | 50% |
|  |  | 100% |
|  |  | 200% |
|  | 25 µmol photons m^-2^ s^-1^ | 50% |
|  |  | 100% |
|  |  | 200% |
|  | 57 µmol photons m^-2^ s^-1^ | 50% |
|  |  | 100% |
|  |  | 200% |
|  | 166 µmol photons m^-2^ s^-1^ | 50% |
|  |  | 100% |
|  |  | 200% |


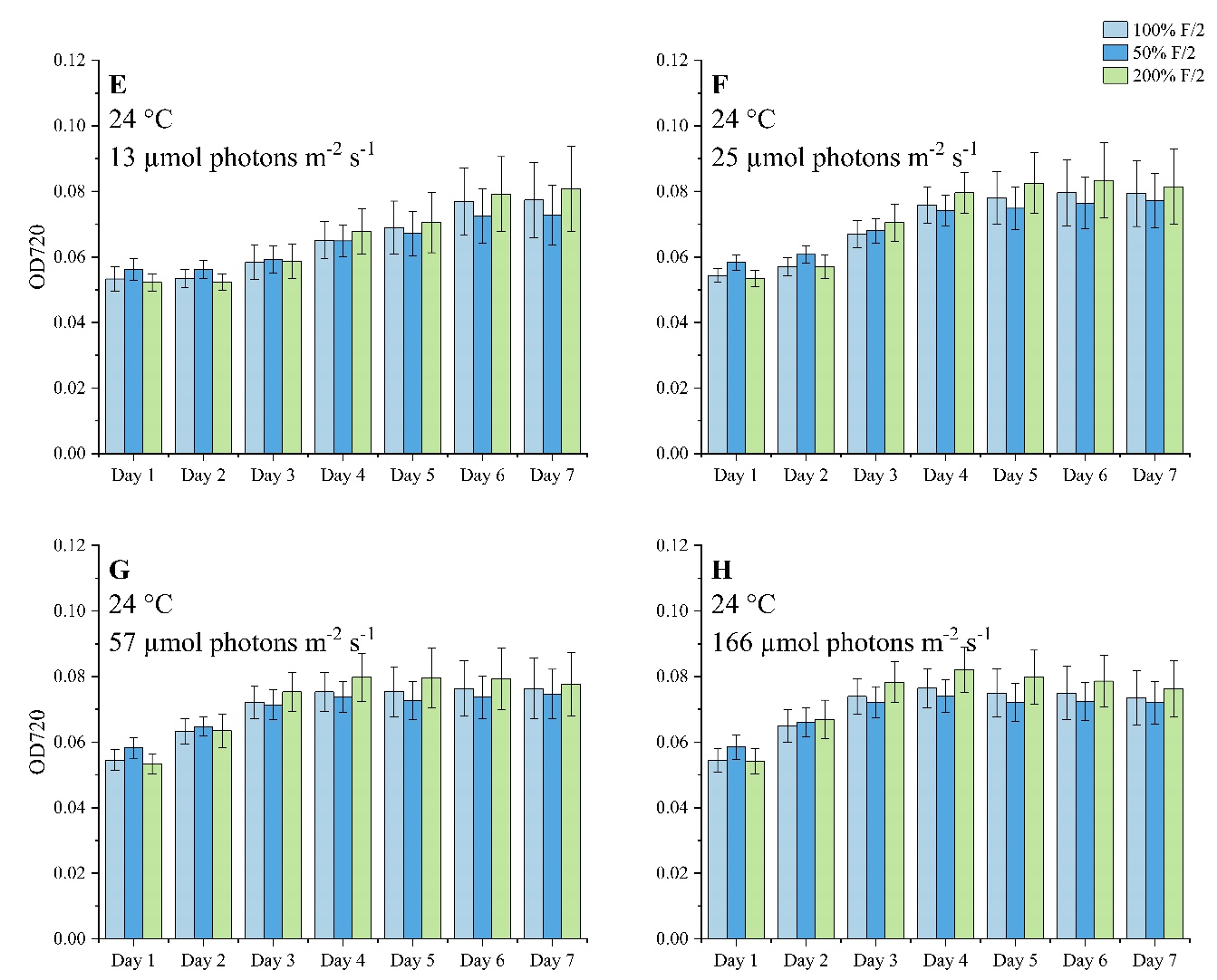

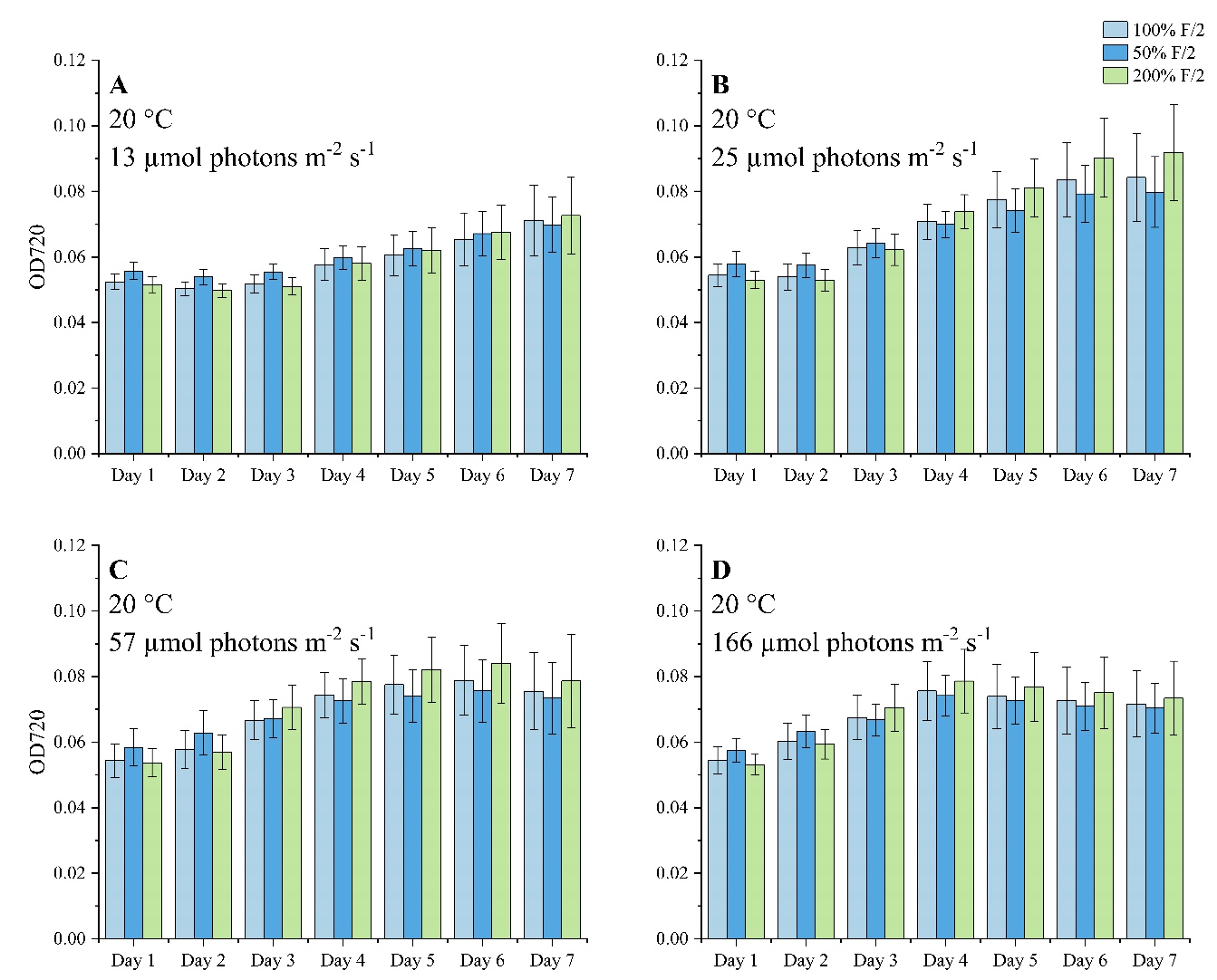

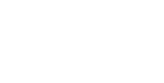


**Supplementary Figure 2.** Histogram of OD720 of *T. weissflogii* culture per environmental condition, as a proxy for biomass accumulation with standard error. Environmental conditions indicated in the top left of each panel. Panel **A-H** are separated by temperature and light combinations with two temperatures (20 °C and 24 °C) and four irradiance levels under each (13, 25, 57, 166 µmol photons m⁻² s⁻¹). Colour is indicative of the nutrient concentration under each environmental condition (100%, 50% and 200%). Daily data is constructed from the average OD720 per day of 192 replicates with error bars representing standard deviation of each group.
